# Supplementary material for: Disrupted PGR-B and ESR1 signaling underlies defective decidualization linked to severe preeclampsia
Source: eLife. 2021 Oct 28;10:e70753. doi: 10.7554/eLife.70753 (PMC8553341; doi:10.7554/eLife.70753)
Supplement: Supplementary file 1. [file elife-70753-supp1.docx]

**Supplementary file 1.** Maternal and neonatal characteristics for endometrial donors.

|  | **sPE (n=24)** | **Control** | |  |  |  |
| --- | --- | --- | --- | --- | --- | --- |
|  |  | **Preterm pregnancy (n=8)** | **Term pregnancy (n=8)** |  | **P* (sPE vs Preterm)** | **P* (sPE vs Term)** |
| Age of the donor (years) | 37.3 (0.8) | 34.9 (2.4) | 37.6 (1.1) |  | n.s. | n.s. |
| Systolic blood pressure (mm Hg) | 164.8 (3.5) | 113.1 (7.7) | 124.0 (6.6) |  | < 0.001 | < 0.001 |
| Diastolic blood pressure (mm Hg) | 99.0 (2.1) | 62.8 (3.0) | 69.4 (2.4) |  | < 0.001 | < 0.001 |
| Proteinuria (mg/dL) | 280.2 (42.5) | 0 or NA | 0 or NA |  | NA | NA |
| Gestational age at delivery (weeks) | 31.9 (0.6) | 33.9 (1.2) | 39.5 (0.4) |  | n.s. | < 0.001 |
| Birth weight (g) | 1651.6 (153.3) | 2403.8 (218.3) | 3200.1 (160.3) |  | < 0.05 | < 0.001 |
| Parity (n) | 1.7 (0.2) | 2.8 (0.7) | 3.4 (0.7) |  | n.s. | < 0.01 |
| Time since last pregnancy (years) | 3.4 (0.3) | 3.3 (1.0) | 4.7 (1.0) |  | n.s. | n.s. |
|  |  |  |  |  |  |  |
| Mean ± SEM * Wilcoxon test |  |  |  |  |  |  |
| NA: Not available |  |  |  |  |  |  |
| n.s.: Not significant |  |  |  |  |  |  |
