# Supplementary material for: Disrupted PGR-B and ESR1 signaling underlies defective decidualization linked to severe preeclampsia
Source: eLife. 2021 Oct 28;10:e70753. doi: 10.7554/eLife.70753 (PMC8553341; doi:10.7554/eLife.70753)
Supplement: Supplementary file 3. [file elife-70753-supp3.doc]

**Supplementary file 3.** RT-qPCR primers list.

| **Sequence Name** | **Sequence** |
| --- | --- |
| PGR_FW | **GTGGGAGCTGTAAGGTCTTCTTTAA** |
| PGR_RV | **AACGATGCAGTCATTTCTTCCA** |
| PGRB_FW | **TCGGACACCTTGCCTGAAGT** |
| PGRB_RV | **CAGGGCCGAGGGAAGAGTAG** |
| IHH_FW | **CTCGCCTACAAGCAGTTCAG** |
| IHH_RV | **CCTGTGTTCTCCTCGTCCTT** |
| MSX2_FW | **ATATGAGCCCTACCACCTGC** |
| MSX2_RV | **GCTTTTCCAGTTCTGCCTCC** |
| ESR1_FW | **ATGTGCCTGGCTAGAGATCC** |
| ESR1_RV | **CAAACTCCTCTCCCTGCAGA** |
