# Supplementary material for: Disrupted PGR-B and ESR1 signaling underlies defective decidualization linked to severe preeclampsia
Source: eLife. 2021 Oct 28;10:e70753. doi: 10.7554/eLife.70753 (PMC8553341; doi:10.7554/eLife.70753)
Supplement: Transparent reporting form [file elife-70753-transrepform1.docx]

***eLife’s* transparent reporting form**

We encourage authors to provide detailed information *within their submission* to facilitate the interpretation and replication of experiments. Authors can upload supporting documentation to indicate the use of appropriate reporting guidelines for health-related research (see [EQUATOR Network](http://www.equator-network.org/%20%20\t%20_blank)), life science research (see the [BioSharing Information Resource](https://biosharing.org/)), or the [ARRIVE guidelines](http://www.plosbiology.org/article/info:doi/10.1371/journal.pbio.1000412) for reporting work involving animal research. Where applicable, authors should refer to any relevant reporting standards documents in this form.

If you have any questions, please consult our Journal Policies and/or contact us: [editorial@elifesciences.org](mailto:editorial@elifesciences.org).

**Sample-size estimation**

- You should state whether an appropriate sample size was computed when the study was being designed
- You should state the statistical method of sample size computation and any required assumptions
- If no explicit power analysis was used, you should describe how you decided what sample (replicate) size (number) to use

Please outline where this information can be found within the submission (e.g., sections or figure legends), or explain why this information doesn’t apply to your submission:

**Replicates**

All details related of sample size are detailed in the ‘material and methods’ section.

No explicit power analysis was used because a particular feature of edgeR functionality, both classic and glm (after glm, TREAT method is used), are empirical Bayes methods that permit the estimation of gene-specific biological variation, even for experiments with minimal levels of biological replication.

- You should report how often each experiment was performed
- You should include a definition of biological versus technical replication
- The data obtained should be provided and sufficient information should be provided to indicate the number of independent biological and/or technical replicates
- If you encountered any outliers, you should describe how these were handled
- Criteria for exclusion/inclusion of data should be clearly stated
- High-throughput sequence data should be uploaded before submission, with a private link for reviewers provided (these are available from both GEO and ArrayExpress)

Please outline where this information can be found within the submission (e.g., sections or figure legends), or explain why this information doesn’t apply to your submission:

Maternal and neonatal characteristics of women with sPE and controls are summarized in supplementary file 1.

Biological and technical variables of interest for controlling confounding effects in the RNA-seq analysis are summarized in supplementary file 2.

Our experimental design randomly split samples into two cohorts, a training set (70%) and a test set (30%) (Figure 1A). Random sampling occurred within each class (sPE and controls), so overall class distribution of the data was preserved. The training set (n=29) was used for the identification of molecular fingerprinting encoding DD in sPE, while the test set (n=11) was used to confirm our findings.

No outliers were encountered. No batch effect was detected.

For quantify gene expression levels by RT-qPCR we analyzed next biological replicates: controls (n=9) and sPE (n=14). For each biological sample, two technical replicates were assessed. For immunofluorescense we used next biological replicates for each group: controls (n=4) and sPE (n=4) for each antibody tested.

The private link to access to high-throughput sequence data is:

<https://www.ncbi.nlm.nih.gov/geo/query/acc.cgi?acc=GSE172381>

Enter token **gjwnaeoyljsphsd** into the box

**Statistical reporting**

- Statistical analysis methods should be described and justified
- Raw data should be presented in figures whenever informative to do so (typically when N per group is less than 10)
- For each experiment, you should identify the statistical tests used, exact values of N, definitions of center, methods of multiple test correction, and dispersion and precision measures (e.g., mean, median, SD, SEM, confidence intervals; and, for the major substantive results, a measure of effect size (e.g., Pearson's r, Cohen's d)
- Report exact p-values wherever possible alongside the summary statistics and 95% confidence intervals. These should be reported for all key questions and not only when the p-value is less than 0.05.

Please outline where this information can be found within the submission (e.g., sections or figure legends), or explain why this information doesn’t apply to your submission:

(For large datasets, or papers with a very large number of statistical tests, you may upload a single table file with tests, Ns, etc., with reference to sections in the manuscript.)

Clinical data and the bar graphs in Figure 5C-5D show mean values and the error bars represent standard deviations. Each dot represent value of each sPE sample analyzed relative to controls. In addition, values are expressed as mean± SEM and significance was coded as *** p<0.001, ** p<0.01, *p<0.05.

Mean values and standard deviations were derived based on all biological and technical replicates of a given sample.

Clinical data were evaluated by non-parametric Wilcoxon test for comparisons between sPE and control samples. Statistical significance was set at p <0.05. Differential expression analysis was performed using the R package edgeR (TREAT method) and significance cut-off was set at False Discovery Rate (FDR) < 0.05 and different fold-changes. Regard gene ontology analysis we use function *goana* in edgeR for multiple testing and we use adjusted p-values (FDR<0.05) for filtering our GO annotations.

**Group allocation**

- Indicate how samples were allocated into experimental groups (in the case of clinical studies, please specify allocation to treatment method); if randomization was used, please also state if restricted randomization was applied
- Indicate if masking was used during group allocation, data collection and/or data analysis

Please outline where this information can be found within the submission (e.g., sections or figure legends), or explain why this information doesn’t apply to your submission:

**Additional data files (“source data”)**

Samples were allocated into sPE or control groups depending on their clinical data. In addition, our experimental design randomly split samples into two cohorts, a training set (70%) and a test set (30%) (Figure 1A). Random sampling occurred within each class (sPE and controls), so overall class distribution of the data was preserved. The training set (n=29) was used for the identification of molecular fingerprinting encoding DD in sPE, while the test set (n=11) was used to confirm our findings.

- We encourage you to upload relevant additional data files, such as numerical data that are represented as a graph in a figure, or as a summary table
- Where provided, these should be in the most useful format, and they can be uploaded as “Source data” files linked to a main figure or table
- Include model definition files including the full list of parameters used
- Include code used for data analysis (e.g., R, MatLab)
- Avoid stating that data files are “available upon request”

Please indicate the figures or tables for which source data files have been provided:

All available data is reported in the supplementary material.

Supplemental file 1 and Supplemental file 2 and Source data 1 were provided for Figure 1. Source data 1 was provided for Figure 2. Source data 1 and Source data 2 were provided for Figure 3. Source data 1 was provided for Figure 4. Supplemental file 3 was provided for Figure 5.

All the R code and data used for analysis and plots generation is available at github repository [**https://github.com/mclemente-igenomix/garrido_et_al_2021**](https://github.com/mclemente-igenomix/garrido_et_al_2021)
